# What We Know and What We Need to Know about Aromatic and Cationic Biogenic Amines in the Gastrointestinal Tract

**DOI:** 10.3390/foods7090145

**Published:** 2018-09-04

**Authors:** Alberto Fernández-Reina, José Luis Urdiales, Francisca Sánchez-Jiménez

**Affiliations:** 1 Departamento de Biología Molecular y Bioquímica, Facultad de Ciencias, Universidad de Málaga, 29071 Málaga, Spain; afernandezreina4@gmail.com (A.F.-R.); kika@uma.es (F.S.-J.); 2 CIBER de Enfermedades Raras & IBIMA, Instituto de Salud Carlos III, 29010 Málaga, Spain

**Keywords:** histamine, serotonin, catecholamines, polyamines, gastrointestinal tract, nutrition, inflammation, gastric cancer, bowel diseases, colon cancer

## Abstract

Biogenic amines derived from basic and aromatic amino acids (B/A-BAs), polyamines, histamine, serotonin, and catecholamines are a group of molecules playing essential roles in many relevant physiological processes, including cell proliferation, immune response, nutrition and reproduction. All these physiological effects involve a variety of tissue-specific cellular receptors and signalling pathways, which conforms to a very complex network that is not yet well-characterized. Strong evidence has proved the importance of this group of molecules in the gastrointestinal context, also playing roles in several pathologies. This work is based on the hypothesis that integration of biomedical information helps to reach new translational actions. Thus, the major aim of this work is to combine scientific knowledge on biomolecules, metabolism and physiology of the main B/A-BAs involved in the pathophysiology of the gastrointestinal tract, in order to point out important gaps in information and other facts deserving further research efforts in order to connect molecular information with pathophysiological observations.

## 1. Introduction

**Figure 1:**
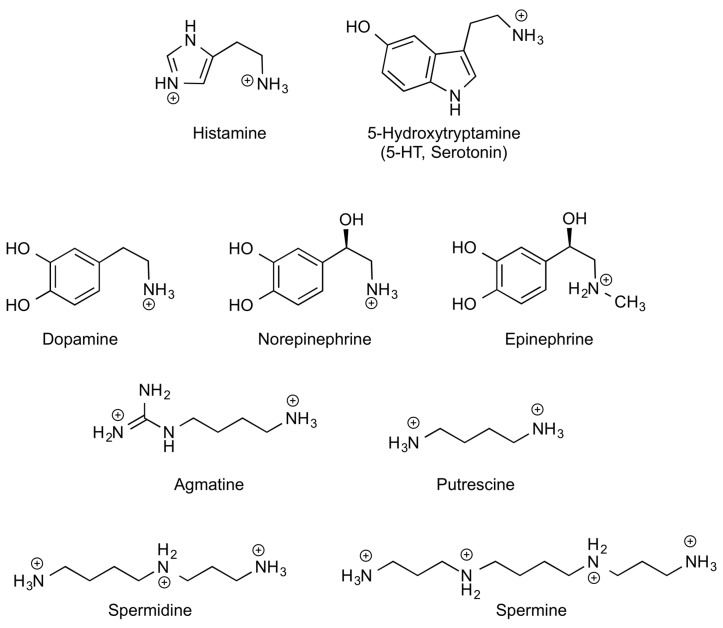
Chemical structures of B/A-BAs in their major forms at physiological pH. Histamine imidazole group is only partially protonated at pH 7 (pI ≈ 6).

Biogenic amines (BAs) are low molecular weight organic compounds synthetized in vivo by decarboxylation of l-amino acids or their derivatives, thus containing one or more amino groups [1]. BAs can be derived from l-basic amino acids, as for instance, histamine (HIS) derived from l-histidine, as well as putrescine (Put), agmatine (Agm), spermidine (Spd) and spermine (Spm) derived from l-arginine or l-ornithine, depending on the organism. Other BAs can also be synthetized from l-aromatic amino acids or derivatives in mammalian tissues, as is the case for serotonin (5-HT) and catecholamines (CAs) that have l-aromatic amino acids such as l-tryptophan and l-tyrosine as their precursors, respectively. Figure 1 shows chemical structures of BAs. Throughout this work, this set of biogenic amines derived from basic or aromatic l-amino acids are abbreviated as BA. We will focus our attention on the role of B/A-BAs, as they are the most important ones in the gastrointestinal context. Another important BA for the central nervous system (CNS), the gamma-aminobutyric acid (GABA), is derived from the amino acid l-glutamate. Many other BAs, outside the scope of this review, can also be synthetized in nature playing different roles along the phylogenetic scale (for instance, tyramine from l-tyrosine and cadaverine from l-lysine, among others) [2,3].

**Figure 2:**
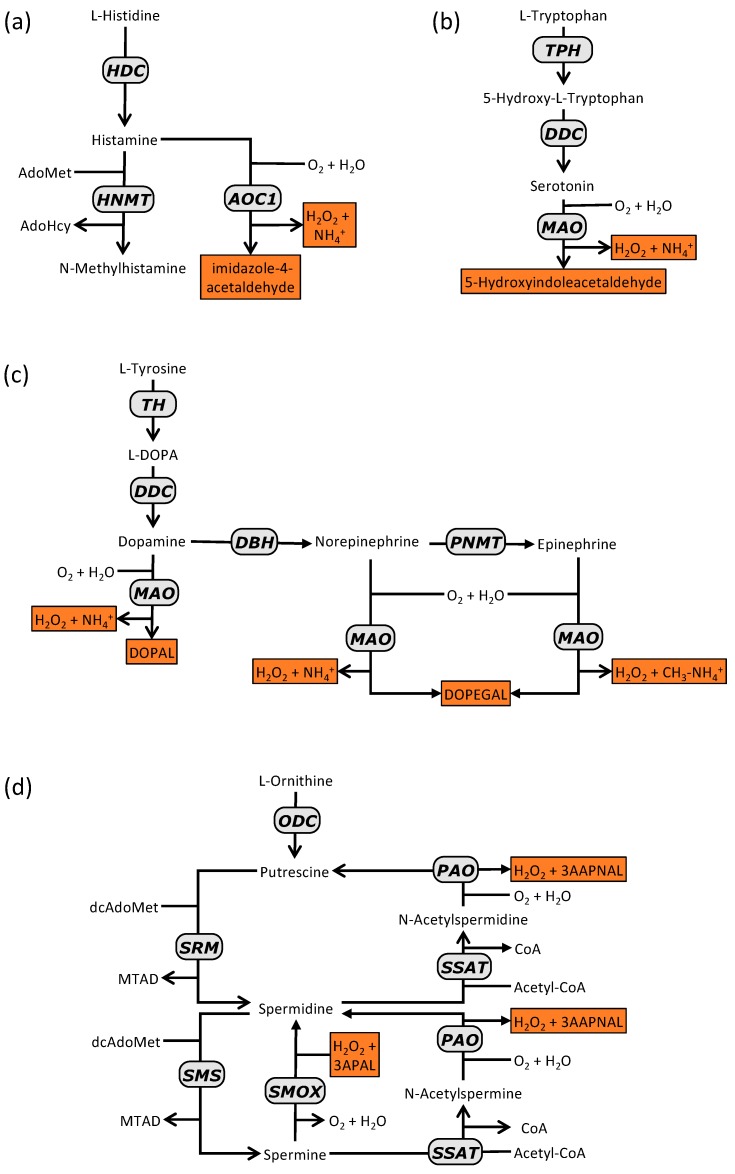
Aromatic and cationic BA synthesis and degradation pathways in mammalian cells. (**a**) histamine; (**b**) serotonin; (**c**) cathecolamines; (**d**) polyamines. Degradation products are depicted in orange boxes. Abbreviations (by alphabetical order): AdoMet, adenosylmethionine; AdoHcy, adenosylhomocysteine; AOC1; CoA, coenzyme A; diamine oxidase; DBH, dopamine β-hydroxylase; dcAdoMet, decarboxylated adenosylmethionine; ADDC, DOPA decarboxylase; DOPA, dihydroxyphenylalanine; DOPAL, 3,4-dihydroxyphenylacetaldehyde; DOPEGAL, 3,4-dihydroxyphenylglycoaldehyde; HDC, histidine decarboxylase; HNMT, histamine *N*-methyltransferase; MAO, monoamine oxidase; MTAD, methylthioadenosine; PAO, polyamine oxidase; PNMT, phenylethanolamine *N*-methyltransferase; SMOX, spermine oxidase; SMS, spermine synthase; SRM, spermidine synthase; SSAT, spermidine/spermine *N*^1^-acetyltransferase; TH, tyrosine hydroxylase; TPH, tryptophan hydroxylase.

All B/A-BA synthetic pathways include the alpha-decarboxylation of l-amino acids with cationic or aromatic side chains, or methylated or hydroxylated amino acid derivatives, as in the cases of 5-HT and CAs, respectively (Figure 2). In mammalian cells, B/A-BA synthesis involves the action of three pyridoxal 5′-phosphate (PLP)-dependent enzymes: ornithine decarboxylase (ODC, EC 4.1.1.17), histidine decarboxylase (HDC, EC 4.1.1.22) and aromatic l-amino acid decarboxylase (or DOPA decarboxylase, DDC, EC 4.1.1.28) [4,5,6]. In some cases, their common names used to derive from the precursor amino acid, as for HIS, that is synthetized from l-histidine, or 5-HT synthetized from l-tryptophan, but it is not a general rule. The metabolic origins of these BAs are shown in Figure 2 and further explained in the following sections.

Expressions of the involved PLP-decarboxylases—ODC, HDC and DDC—are cell-specific events, therefore linked to cell-specific developmental programs, for which we still ignore many involved factors. Both mammalian HDC and DDC share a high degree of homology; however mammalian ODC has a different evolutionary origin [7,8]. Nevertheless, all of them could compete for the cofactor PLP, in cases of vitamin B6 deficit or altered hepatic PLP metabolism (for instance, during aging [9]).

Another common fact is that BA degradation in vivo involves the action of amino oxidases. These reactions produce aldehydes (sometimes very toxic ones) and H_2_O_2_. A high oxidase activity could therefore cause local ROS and/or toxic aldehyde elevations. Amine oxidase specificities for each B/A-BA will be mentioned below. There are two families of amine oxidases, copper- or flavine-dependent oxidases [10,11]. These enzymes can be extra- or intracellular located and they also differ in the amine substrate specificities. For instance, the copper-dependent diamine oxidase (AOC1 or DAO, EC 1.4.3.22) can accept both HIS and Put as a substrate; these BAs come from different synthesis pathways. DDC products also share MAO activities (EC 1.4.3.4) [12]. Thus, degradation is also a process in which different BA metabolic pathways can eventually be confluent in the same physiological context.

In the following subsections, we will focus on descriptive overviews of the different B/A-BA specificities of their respective metabolic pathways and physiological functions in the gastrointestinal tract (GIT) system. It is a very complex physiological scenario still unveiled or confusing in many aspects. However, it is well known that BA metabolism in GIT can be highly decisive for health and quality of life, as occurring in the other physiological contexts mentioned above, therefore also deserving further biochemical and cellular research efforts to reach more efficient translational actions.

From a biochemical point of view, BAs were considered to be a part of secondary metabolism for many years, and consequently neglected in many general biochemistry textbooks. However, evidence has accumulated revealing important roles of these metabolites in mammalian pathophysiology. For instance, it is well known that HIS is an important mediator of the immune system, as well as a key biomolecule for correct gastric function [13,14,15] (Table 1). Nowadays, we can say that they are very important for human homeostasis, as they play important roles in the most important human physiological functions (neurotransmission, defence, digestion and nutrition, growth, apoptosis, and reproduction). Consequently, impairments in their metabolic (including signalling) pathways are related to many different pathological phenotypes and diseases.

BAs can be synthesized de novo by specific mammalian cell types, but can also have an exogenous origin [19]. Microbiota, as well as microorganisms taking part in food processing or contamination, can produce biogenic amines from dietary amino acids at different rates and with different structures to those synthetized by human cells, which can have physiological effects; for instance, the decarboxylation product of l-arginine, Agm [20]. Its endogenous synthesis is, at least, controversial [21]. BAs are also present in a huge variety of drinks and foods, especially those in which microbial activity takes place during storage or preparation, sometimes with negative consequences for human health; for instance, toxicity due to HIS overproduction in contaminated seafood (i.e., by *Morganella morganii* sp.) [22] or high levels of amines in cold cuts and fermented foods (lactic products, fermented vegetables, wine, beer, etc.) [23,24]. In addition, BAs could form carcinogenic nitrosamines in the presence of nitrites during food processing [25].

A full characterization of the physiological effects of exogenous BAs has always faced two big handicaps: the multiple difficulties to evaluate the degree in which dietary amines are absorbed by gut epithelium, and the complexity of the characterization of the BA metabolism capacities of our particular microbiota, as this factor can induce important changes in the BA concentrations available to gut epithelium.

The importance of B/A-BAs in our digestive systems has been observed throughout the 20th century. In spite of all these valuable pathophysiological data (thousands of indexed publications) available, many gaps in molecular information still exist with regard to the mechanisms involved in each case, delaying the progress towards more personalized and accurate solutions for digestive-related pathologies [26]. As a research group working on several Systems Biology initiatives [27,28] and BAs [1,29,30], our hypothesis is based on the concept that integration of information can reveal emergent information, offering light to new hypothesis and translational actions. As far as we know, there is no recent similar review devoted to gathering biochemical and pathophysiological information on B/A-BAs in the GIT. Thus, the major aim of this work is to present an overview of the known facts of biochemical and pathophysiological information on B/A-BAs in the GIT context. The objective is to point out interesting facts deserving further research in order to eliminate gaps of molecular information currently blocking or delaying translational possibilities for prevention, diagnosis, and/or intervention of gastrointestinal diseases.

## 2. Histamine Biochemistry and Physiology

### 2.1. Histamine Synthesis

HIS was one of the first discovered low molecular weight (111 Da) immune mediators at the beginning of the last century [17,31,32]. Its precursor, the semi essential amino acid l-histidine, can be at least partially endogenous or derived from dietary proteins [33]. In mammalian cells, HIS synthesis occurs by decarboxylation of l-histidine, which is catalysed by the enzyme named l-histidine decarboxylase (HDC) (Figure 2a). This activity is carried out by PLP-dependent enzymes in both Gram-negative bacteria and Metazoa [34]. However, in Gram positive bacteria potentially present in intestines (for instance, *Lactobacillus* sp.), the reaction is catalysed by a non-homologous pyruvoil-dependent enzyme [5,34,35]. In human tissue, HDC is only expressed in a short list of cell types. Among them, several immune differentiated cells (mast cells and basophils) [36,37,38], histaminergic neurons [18,39], and gastric enterochromaffin-like cells (ECL cells) [40] are able to both synthetize and store HIS. Transformed HIS producing cells can preserve or even increase their HIS-producing capacity, as in the case of malignant mastocytosis and several types of gastric cancer cells [41,42]. Other cells (for instance, macrophages, eosinophils, and platelets) can also synthetize HIS to any extent, being unable to store it in specialized vesicles [31,35].

A big gap of information still exists on mechanisms controlling cell type-specificities with respect to HDC expression, but it seems to be clear that epigenetic events play important roles (i.e., methylation/demethylation of CpG islands present in mammalian HDC gene promoter) [43,44]. It has been observed that HDC gene expression can increase in response to several stimuli such as gastrin, estrogens, or several interleukins (ILs) as IL-1, IL-3, IL-12 or IL-18. It depends on the specific receptors expressed by the target HIS-producing cells [35,45].

Alternative splicing events have been observed during mammalian (and human) HDC expression in HIS-producing cells [46,47]. The meaning of these aberrant messengers is still unknown. As the active protein is a dimer and taking into account that dimerization involves interaction of both N-terminus [34], some of the truncated sequences could act as natural HDC inhibitors.

In addition, the protein needs to be processed to reach the active conformation and is a very unstable enzyme [48,49,50,51,52,53]. Regulation of the enzyme processing and turnover can be important as a determinant of active HDC levels. However, HDC processing and maturation is a process not fully characterized. It seems to be clear that the monomer mature form must correspond to a 53–63 KDa fragment of the N-terminus of the primary translation product [49]. Nevertheless, the precise sequence of this fragment in vivo is not yet known.

The action mechanism of mammalian PLP-dependent l-amino acid decarboxylases has been previously described [54,55]. Briefly, it involves two transaldamination reactions from the PLP-enzyme complex to the l-amino acid-enzyme complex, which is decarboxylated in the substrate α-carboxylic group to form a covalent amine product-enzyme complex. This last complex suffers a second transaldimination reaction with PLP to recover the initial PLP-enzyme complex, thus releasing the amine product. Important changes in the global decarboxylase conformation have been observed for both mammalian HDC and DDC during catalysis [56,57]. The quaternary structure of HDC and DDC only differs in tautomeric forms of intermediates along the reaction, most probably due to slight differences in the active dimer conformation [54,55]. In fact, both enzymes can share substrates (i.e., l-histidine, but with different affinities) and inhibitors (for instance, epigallocathechine-3-gallate). This fact needs to be taken into account for design of specific inhibitors of any of these activities with pharmacological purposes.

### 2.2. Exogenous Histamine Synthesis

Important quantities of HIS can be present in some natural products, such as oranges or tomatoes. Fermented products (cheese, alcoholic drinks, fermented vegetables and fish) can also contain high quantities of HIS (and other BAs), as a result of the metabolic properties of the living organism involved in each case. Contamination during food processing or storage can also allow undesirable growth of HIS-producing microorganisms. Many efforts of EU COST actions (i.e., COST Action 917, 922 and BN0806) have been devoted to the study and control of BA levels in foods (see for instance, [58,59]) However, it is a very complex subject with many variables and a lot of uncertainty with regard to amine absorption mechanisms and traceability. This issue requires applying more holistic approaches, and the use of high-throughput technologies, in order to efficiently translate the knowledge to both new general nutritional recommendations and personalized diets.

In addition, GIT microbiota species can synthetize HIS (and many other BAs) by using PLP- or pyruvoyl-dependent enzymes. Thus, microbiota characterization should also be considered during personalized medicine initiatives of patients affected by BA-related diseases [26].

### 2.3. Histamine Degradation

In human tissues, HIS can be degraded by two different pathways (Figure 2a). The first one involves the intracellular *N*-methylation of HIS in its imidazole group catalysed by histamine *N*-methyl transferase (HNMT, EC 2.1.1.8) [60]. It is an ubiquitous enzyme expressed in liver and also in intestinal mucosa in a minor extent [61,62]. Its product, N-tele methyl-histamine, is a substrate of monoamine oxidase (MAO), which produces *N*-methylimidazole acetaldehyde. Finally, the enzyme aldehyde dehydrogenase (AD, EC 1.2.1.5) reduces this metabolite to *N*-methylimidazole acetic acid. This seems to be the major pathway in the brain [63]. However in GIT, the main pathway for HIS degradation involves the direct HIS oxidation by the action of human DAO producing imidazole acetaldehyde [35]. It is a copper-containing glycoprotein associated with cytosolic membrane and expressed in the stomach, duodenum, small intestine, and colon [61,62]. It can be released from membranes of their producing cells and is active in human serum [64]. It is also known as amyloride-binding protein-1 (AOC1) and histaminase.

### 2.4. Histamine Transport and Storage Mechanisms

In mammalian cells, HIS can be transported into epithelial cells throughout organic cations transporters (OCT 2 and 3), and the plasmatic membrane monoamine transporter (PMAT). OCT 2 and 3, and PMAT are located in the basolateral plasmatic membranes. OCT 3 has also been located in the luminal membranes of bronchial and small intestine epithelial cells [35,65].

Inside the cell, HIS can be transported through endosomal membranes by using the vesicular amine/proton antiporter systems named vesicular monoamine transporter 2 (VMAT2 or SLC18A2), which is also able to transport other monoamines such as DA, norepinephrine and 5-HT, and can be modulated by drugs such as amphetamines and cocaine [66].

With respect to storage, as mentioned previously, only a few cell types are able to store HIS. The major HIS-storing cells in a human body are mature mast cells, which can accumulate HIS, as well as 5-HT and even PAs into secretory granules, most probably derived from *trans*-Golgi vesicles [67]. Mast cells are infiltrated into mammalian epithelia, including GIT epithelia. Other important HIS storing cells in GIT are gastric ECL cells [68].

Three different mechanisms have been described for HIS secretion [35]:

1. Mast cell degranulation by immune stimuli. The presence of specific antigens induces IgE synthesis, inducing a high affinity binding between the specific IgE and IgE receptor known as FcεRI. This high affinity complex induces degranulation after further expositions to the antigen.
2. Cytokines can also induce degranulation. It is mediated by vesicular trafficking events involving fusion and/or content interchange between secretory granules and vesicles driven to exocytosis.
3. Constitutive HIS leakage due to non-active transport through cytosolic membranes or *trans*-Golgi vesicles driven to exocytosis.

### 2.5. Histamine Signalling and Physiological Functions

HIS could be considered the most pleiotropic amine, as it is involved in a wide spectrum of physiological processes concerning the most important function for a human being. It is a well-known immune mediator, as well as a neurotransmitter, thus involved in the most complex and still not fully characterized physiological capabilities. It also plays a key role in gastric acid secretion, and has also been described as a cell proliferation modulator, nutrition and cell proliferation being two essential functions for life [13].

HIS effects in different physiological scenarios are elicited by different HIS receptors. Four specific HIS receptors have been detected so far, namely H_1_R, H_2_R, H_3_R and H_4_R. All of them are members of the G-protein coupled receptor family. Their expressions are cell-type dependent and the elicited signals sometimes contradictory. Nevertheless, all of them somehow participate in GIT functions and homeostasis. Table 2 summarizes their specific characteristics.

H_1_R and H_2_R are the most ubiquitous receptors along the GIT. H_1_R, H_2_R and H_3_R are located in gastric mucosa and their affinities for HIS are dependent on the expressed isoforms. H_4_R can be expressed by inflammatory cells, as well as peripheral neurons associated with GIT. Specific HIS actions throughout the GIT are summarized below. H_4_R was the most recent HIS receptor discovered, and is still not fully characterized, in spite of the multiple efforts made by international research groups [69]. Nevertheless, insights point out to its importance in GIT physiology, thus encouraging new actions to decipher this still veiled but important information for GIT pathophysiology. In fact, the receptor is proposed to be involved in gastric acid secretion, gastric mucosa defence, intestinal motility and secretion, visceral sensitivity, inflammation, immunity and gastric and colorectal carcinogenesis [70].

#### 2.5.1. Histamine and Acid Gastric Secretion

Gastric acid secretion is regulated by different positive stimuli, such as acetylcholine, HIS and gastrin, and inhibitors such as somastostatin. Acetylcholine is a neurotransmitter coming from enteric neurons. HIS, gastrin and somatostatin are secreted by different endocrine cells infiltrated in GIT mucosa; they include ECL cells, G cells and D cells, respectively [71].

**Figure 3:**
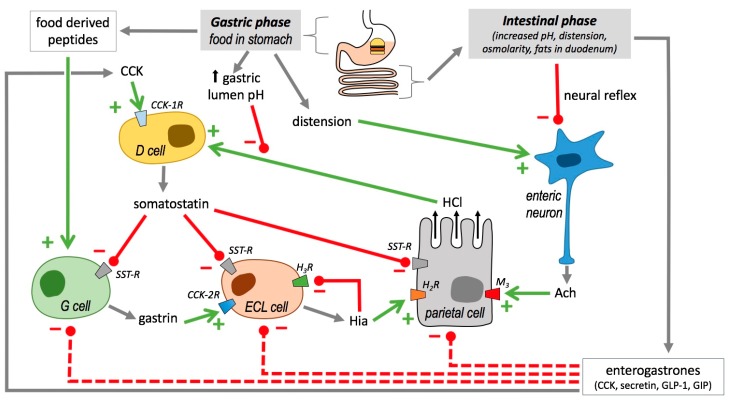
Balance between stimuli and inhibitors of gastric secretion. CCK, cholecystokinin; CCK_n_R, different types of cholecystokinin receptors (1 or 2); D cell, somatostatin-releasing cell; ECL cell, enterochromaffin-like cell; G cell, gastrin-producing cell. GLP-1, glucagon-like peptide; GIP, gastric inhibitory polypeptide; H_n_R, different types of histamine receptors; M_3_, muscarinic acetylcholine receptor type 3; SST-R, somatostatin receptors. Products are represented by grey arrows, activations by green plus symbols and arrows, and inhibitions by minus symbols and red bars.

Figure 3 is a scheme of the balance between stimuli and inhibitors of gastric secretion. Briefly, on the one hand, binding of acetylcholine (from enteric neurons) to specific receptors stimulates parietal cells to secrete HCl; as well as gastrin (from gastric epithelium G cells), which binds to the cholecystokinin receptor 2 (CCK2 receptor) of ECL cells, thus inducing HIS secretion. As mentioned before, HIS is a stimulus for HCl secretion by parietal cells through the signalling pathway elicited by H_2_R. On the other hand, circulating cholecystokinin (CCK) binds to CCK1 receptors of gastric D cells, thus stimulating somatostatin secretion [71]. Somatostatin directly inhibits acid secretion by parietal cells, as well as both HIS and gastrin secretion [72].

The balance between stimuli and inhibitors change throughout different phases involved in the process, including an intracranial phase, a gastric phase, and an intestinal phase. In the next paragraphs, we will focus on phases directly related to GIT.

In the gastric phase, the presence of food in the stomach induces acid gastric secretion by three different ways (Figure 3): the stomach distention caused by the food is detected by mechanoreceptors, which in turn induces neuronal reflexes for acetylcholine production; food derived-peptides and amino acids stimulate gastrin secretion by G cells; food increases gastric lumen pH, which is an inhibitory signal for somatostatin secretion [72].

When chyme reaches the duodenum, negative feedback mechanisms operate to reduce acid secretion (Figure 3). On the one hand, neuronal reflexes are activated, therefore blocking acetylcholine induced HCl secretion. On the other hand, in enteroendocrine cells, the synthesis of somatostatin synthesis activators (i.e., CCK, secretin, glucagon-like peptide and gastric inhibitory polypeptide) [73] are also promoted in different enteroendocrine cell types, which finally lead to gastrin, HIS and HCl secretion inhibition (Figure 3).

It has been proposed recently that HIS could also inhibit its own secretion through binding to H_3_R present in ECL cells membranes [17]. Acting through other receptors, HIS has also been proposed as involved in the gastric vasodilatation and reactive hyperaemia produced in response to acid challenge (through H_1_R), and the modulation of the gastric mucosal defence, the enteric neurotransmission and the feedback regulation of HIS release (through H_3_R, and maybe also through H_4_R) [73,74]. Nevertheless, the precise roles of H_4_R in gastric physiology are still controversial [75].

#### 2.5.2. Histamine and Immune Response in Gastrointestinal Tract

As mentioned before, mast cells (and basophyls) are the major producers of HIS. In these cells, HIS release can be induced by IgE, but also by cytokines, neuropeptides, growth factors, free radicals and anaphylotoxins [17], many of them potentially present in the GIT. Other eventually HIS-producing, but not HIS-storing cells, like lymphocytes, fibroblasts and macrophages are also present and interact with GIT epithelia [76,77].

HIS modulates immune response mainly through H_1_R, H_2_R and H_4_R, depending on the receptor type expressed in each immune cell type. HIS elicited immune actions include the capability to modulate expression and/or activity of many cytokines and the complement system [78,79,80]. Again in turn, a cross regulation between cytokines and HIS seems to control GIT functions, as it has been proven that several cytokines such as TNF-α, IL-1 and IL-6 modulate HIS synthesis and secretion. Several components of complement systems like C3a, C4a and C5a (anaphylotoxins) have the capability to induce HIS release from mast cells and basophils [17]. During the last decade, interest of the role of H_4_R in the GIT context has increased [81].

## 3. Serotonin Biochemistry and Physiology

### 3.1. Serotonin Synthesisn 

5-HT is an l-tryptophan-derivative (Table 1). Meat, milk and fruit are the major sources of the essential amino acid precursor [82]. About 95% of the total 5-HT content in a human body is synthetized by GIT-associated cells (approximately 9/10 by intestinal enterochromaffin-cells (EC), and 1/10 by serotoninergic neurons located in the myenteric plexus. Only 5% of the 5-HT content in a human body is estimated to be synthetized in CNS [83].

The biosynthetic 5-HT pathway begins with the hydroxylation of the indole moiety C5 (Figure 2b) catalysed by the enzyme tryptophan hydroxylase (TPH, EC 1.14.16.4). It is a tetrameric non-heme iron-dependent monooxygenase that uses l-Trp and oxygen as substrates and tetrahydrobiopterin (BH_4_) as the cofactor. The reaction occurs as two sequential half reactions: a reaction between the active site iron, oxygen, and the tetrahydropterin to produce a reactive Fe^IV^O intermediate and the hydroxylation of the amino acid by Fe^IV^O [84]. Two isoforms have been detected for this enzyme. TPH-2 expression is almost exclusive for neurons, and TPH-1 is expressed in other 5-HT-producing cell types [85].

The TPH product, 5-hydroxy-l-tryptophan (5-HTP), is the substrate of DDC that produces the amine 5-HT by decarboxylation of the 5-HTP α-carbon. This enzyme also decarboxylates other aromatic l-amino acids or derivatives; for instance, l-dihydroxyphenylalanine (l-DOPA) to produce DA [86]. In addition, it is also able to catalyse other reactions under different environmental circumstances or specific mutations (for instance, half-transaminations and oxidative deaminations) [87,88]. The mammalian enzyme is also a PLP-dependent enzyme, highly homologous to mammalian HDC, as mentioned above [26,89]. In fact, it is able to accept l-His as a substrate but with a much lower affinity than for 5-HTP or DOPA; however, the human DDC gene lacks the sequence encoding the carboxy-terminal fraction present in mammalian HDC, which is involved in mammalian HDC sorting to endoplasmic reticulum and activation [49]. This could suggest a different intracellular location for both enzymes. Mammalian HDC and DDC share the catalytic mechanism explained above for HDC [54,90]. However, at least in the case of the purified recombinant wild proteins, mammalian DDC seems to be a more efficient enzyme according to their respective catalytic constant values obtained in silico and in vitro [54,55]*.* In the case of DDC, slight modifications of the catalytic site environment seem to induce important changes in catalytic constant (*k_cat_*) values [90]. Both enzymes (DDC and HDC) also share other structural properties related to enzyme stability and catalysis. For instance, the presence of PEST regions in the N-terminus of the monomers and a highly labile flexible loop, which is essential for conformation changes of the enzymes during PLP binding and for catalysis itself [6,53,88]. In the case of 5-HT biosynthesis, the limiting step is not decarboxylation but TPH activity.

It is noteworthy that HIS and 5-HT synthesis exhibit antagonist time-course patterns during differentiation of mouse bone marrow derived cells to mast cells in vitro, as well as opposite responses to PA inhibitors [67]. These results suggest a sort of regulatory coordination among all of these amine biosynthetic processes, which are not fully characterized yet, but should be taken into account in all of the pathophysiological scenarios where synthesis of these amines can be confluent, as GIT is.

### 3.2. Serotonin Degradation

5-HT degradation is catalysed mainly by any MAO activity (Figure 2b). MAO catalysis requires FAD as the cofactor to carry out an oxidative deamination of 5-HT, thus producing hydrogen peroxide and 5-hydroxy-3-indolacetaldehyde, which is rapidly processed to 5-hydroxy-3-indolacetic acid by the action of AD [91]. Human genome contains two different genes encoding MAO activities, namely MAO-A and MAO-B [12]. Both proteins are located in the external mitochondrial membrane [92]. MAO-A has a higher affinity by 5-HT as well as a wider expression spectrum. However, MAO-B is the only one detected in serotoninergic neurons [91]. Both MAO isozymes are expressed in GIT [61,62,93].

### 3.3. Serotonin Transport and Storage Mechanism

In GIT, 5-HT is mainly produced and secreted by the neuroendocrine enterochromaffin (EC) cells, located alongside the intestinal epithelium lining the lumen of the digestive tract. Recently, it was described that the sodium channel Na_v_1.3 plays an important role for EC excitability and 5-HT release [94]. Once 5-HT is secreted by EC cells and binds to the specific receptors of the surrounding cells, it is removed from the interstitial space by the sodium-dependent 5-HT transporter (SERT), also named as the solute carrier family 6 member 4 (SLC6A4), which is expressed by GIT epithelial cells. SERT is a protein with 12-transmembrane fragments, which is able to transport 5-HT by a Na^+^/K^+^- and Cl-dependent mechanism [83,95]. Inside the GIT epithelial cells, 5-HP is rapidly degraded by MAO activity, as explained above [83,95,96].

In addition, postprandial 5-HT can also enter systemic circulation and is absorbed by platelets. Actually, most of the circulating 5-HT is stored in platelets, as these cells also express 5-HT transporters. SERT is negatively regulated by activation of tool-like receptors and several pro-inflammatory cytokines. On the contrary, other anti-inflammatory cytokines, such as IL-10, increase transporter activity. Treatment with specific SERT inhibitors leads to an increase of free 5-HT content, thus empowering 5-HT effects, not only in GIT but also in CNS [83].

### 3.4. Serotonin Signalling and Physiological Functions

It is well known that 5-HT has also been involved in very complex physiological processes such as being an essential neurotransmitter and paracrine molecule for brain-intestine crosstalk, commonly known as gut-brain axis [97]. The amine is involved in modulation of body temperature and circadian rhythm [98,99], as well as in cardiovascular activity, morphogenesis and cell proliferation [100,101]. In the GIT context, it has been described as a gastric motility and secretion, a nutrient absorption regulator and an immunoregulatory compound. Consequently, dysfunctions in 5-HT metabolism usually have very important negative consequences on human physiology including gut-brain communication [82,102]. 5-HT also elicits both motor and sensitive responses in the intestine by binding to different receptors expressed by mesenteric and mucosal neurons (Table 3).

#### 3.4.1. Regulation of GIT Smooth Muscle Contraction and Relaxation

**Figure 4:**
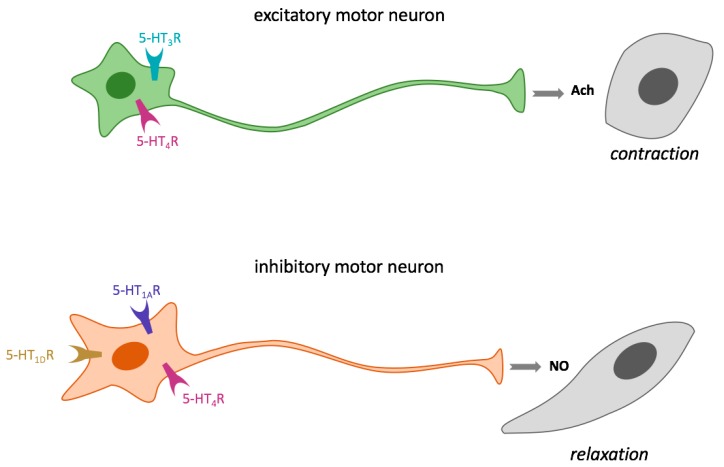
Regulation of GIT smooth muscle contraction and relaxation through 5-HT receptors. AcH, acetylcholine; 5-HT_1A_R serotonin receptor type 1A; 5-HT_1D_R; serotonin receptor type 1D, 5-HT_3_R; serotonin receptor type 3, 5-HT_4_R; serotonin receptor type 4.

5-HT is a regulator of both intestinal smooth muscle contraction and relaxation through the activation of enteric excitatory motor neurons and intrinsic inhibitory neurons, respectively [83,104]. The amine can bind 5-HT_3_R and 5-HT_4_R of excitatory cholinergic motor neurons, thus inducing acetylcholine release and smooth muscle contraction. However, 5-HT binding to 5-HT_4_R, 5-HT_1A_R, and/or the badly characterized 5-HT_1D_R, present in inhibitory nitrergic motor neurons induces nitric oxide (NO) synthesis and consequently smooth muscle relaxation (Figure 4). In addition, 5-HT also participates in gastric muscle motility regulation [83,105].

#### 3.4.2. Mucosal Sensory Transduction

EC cells secrete 5-HT in response to intraluminal pressure. The released amine can stimulate both the intrinsic primary afferent neurons (IPANs) located in submucosal and myenteric plexus and the extrinsic afferent neurons (vagal and spinal), through their binding to different receptors: 5-HT_3_R, 5-HT_4_R, 5-HT_7_R; and 5-HT_1D_R [83]. 

On the one hand, submucosal neurons release both acetylcholine and calcitonin gene-related peptide; however, myenteric neurons only release acetylcholine. Both neuron types are involved in the modulation of intestinal motility, secretion, and vasodilatation. Thus, submucosal neurons initiate peristaltic and secretory reflexes, and myenteric neurons start migratory contractions. On the other hand, spinal afferent neurons transmit signals related to digestive reflexes, satiety, and pain from the intestine to the CNS. 

In addition, some authors claim an important role of neuronal 5-HT in promotion of development/survival of some classes of late-born enteric neurons, including dopaminergic neurons, which appear to innervate and activate the adult enteric nervous system [104].

#### 3.4.3. Serotonin and Immune Response in GIT

It has been reported that 5-HT can also elicit pro-inflammatory responses in GIT that involve different transduction pathways most probably started by the amine binding to the 5-HT receptors expressed by dendritic cells located in *lamina propria*. Recently, this immuneregulatory role of 5-HT in GIT has been the subject of very relevant reviews on the topic [95,102].

## 4. Biochemistry and Physiology of Catecholamines

### 4.1. Synthesis of Catecholamines

DA, and their derivatives noradrenaline/norepinephrine and adrenaline/epinephrine, are the most important cathecolamines for human physiology (Table 1). All of them are synthetized from l-phenylalanine or l-tyrosine mainly from diet (Figure 2c). l-phenylalanine can be the substrate of phenylalanine hydroxylase (PAH) to produce l-tyrosine. The limiting step for DA synthesis is the enzyme tyrosine hydroxylase (TH, tyrosine 3-monooxygenase, EC 1.14.16.2), which introduces a hydroxyl group in the meta position of the cathecol ring of l-tyrosine to obtain l-3,4-dihydroxyphenylalanine or l-DOPA). This reaction requires Fe^2+^, the cofactor BH_4_ and O_2_. TH is mainly expressed in neuroendocrine cells in both soluble and membrane bound forms. It is a highly stereo specific enzyme, although it can also act on l-phenylalanine [82,106]. Up to four different alternative TH mRNA spliced forms have been detected. The meaning of this variability is still uncertain [106].

l-DOPA is a substrate of the DDC mentioned in the previous section, producing DA. In fact, it has also been named as dopa decarboxylase in the literature [6,90]. La AADC or DDC is a ubiquitous enzyme expressed by cell types located in different organs like the adrenal medulla, kidney, liver, GIT and brain [61,106].

In addition to the above-mentioned pathway, there are two other alternative ways to produce DA (not shown in Figure 2). One way, l-tyrosine, can also be decarboxylated by AADC to produce tyramine, which is hydroxylated by a member of the cytochrome P450 family (family 2, subfamily D, or CYP2D). Nevertheless, l-phenylalanine can also be decarboxylated by AADC to produce phenyltyramine, which can be converted to DA by CYP2D [92].

In several peripheral tissues (mainly in adrenal medulla), two other further reactions can take place to produce norepinephrine and epinephrine. Firstly, the action of the enzyme dopamine β-hydroxylase (DBH, dopamine β-monooxygenase, EC 1.14.17.1) produces norepinephrine. This oxidase requires ascorbic acid as the electron donor. It is a highly antigenic homotetramer (Mr around 290 kD) with low substrate specificity [106].

Finally, the enzyme phenylethanolamine *N*-methyltransferase (PNMT, EC 2.1.1.28) catalyses the *N*-methylation of norepinephrine to produce epinephrine (Figure 1c). PNMT is a cytosolic enzyme that uses *S*-adenosylmethionine (SAM) as the methyl donor. It has a low substrate specificity that allows it to carry out the beta carbon methylation of a variety of amines. Its expression is mainly but not exclusively restricted to the suprarenal glands [106,107].

### 4.2. Degradation of Catecholamines

As occurring with other BAs, CAs can be the subject of oxidative deamination catalysed by MAO, thus producing H_2_O_2_ and aldehydes, 3,4-dihydroxyphenylacetaldehyde (DOPAL) from DA, and 3,4-dihydroxyphenylglycoaldehyde (DOPEGAL) from epinephrine and norepinephrine (Figure 2c). Both are instable compounds rapidly oxidized to dihydroxyphenylacetic acid and 3,4-dihydroxymandelic acid, respectively, by the action of AD [108,109]. Alternatively, the enzyme aldehyde reductase (AR, EC 1.1.1.21) can reduce DOPAL to 3,4-dihydroxyphenylethanol, and DOPEGAL to dihydroxyphenylglycol. The lack of a beta-hydroxyl group in DOPAL favors its oxidation by AD. Conversely, the presence of the β-hydroxyl group in DOPEGAL makes it a better substrate of AR [108].

Nevertheless, the major product of norepinephrine degradation in humans seems to be vanillylmandelic acid (VMA), produced mainly by a pathway that requires the consecutive actions of MAO, AR, catechol O-methyltransferase (COMT, EC 2.1.1.6) alcohol dehydrogenase (ADH, EC 1.1.1.1) and AD [108]. COMT also uses SAM as the methyl donor. Two different isoforms are encoded by a unique gene, the cytosolic isoform being the one present in glia and peripheral organs (for instance, liver and kidney) (not shown in Figure 2).

DA and their derivatives can be converted into other molecules in CNS and peripheral tissues before being excreted. The activity phenolsulfotransferase (EC 2.8.2.1) is able to produce dopamine-3-*O*-sulfate and dopamine-4-*O*-sulfate by transferring a sulfate group of 3′-phosphoadenosine-5′-fosfosulfate to any hydroxyl groups of the catechol (preferably to dopamine-3-*O*-sulfate). Moreover, the enzyme uridine diphosphoglucuronosyltransferase (EC 2.4.1.17) transfers glucuronic acid from UDP-glucuronic acid to both hydroxyl groups of the dopamine catechol ring. This enzyme is linked to the reticulum endoplasmic membrane [109].

### 4.3. Signalling and Physiological Functions of Catecholamines

CAs act as both neurotransmitters and hormones, depending on their targets in different tissues/organs [110]. In CNS, they are mainly involved in motor and emotional control, cognition, and memory [111].

In peripheral tissues, they also play important roles as modulators of blood pressure and renal excretion, as well as in the immune system and GIT functions [112]. DA receptors are G protein-coupled receptors (GPCRs) classified into 5 types (D1–D5) [113]. Heterodimerization among the subtypes and with other receptor type monomers have been reported, which results in a very complex cell-dependent signalling network. In addition, DA can also elicit physiological signalling through G-protein independent mechanisms (i.e., ion channels, tyrosine kinases and even arrestins [113].

Epinephrine and norepinephrine preferably bind to adrenergic receptors or adrenoceptors (all GCPRs) that are classified as α (1a,1b,1d and 2a,2b,2c) and β (1–3) subtypes [82,114]. In the intestine, the main epinephrine receptors are α1 and β2. However, in the case of norepinephrine, they are α1 and α2 (as well as β2, but in a minor degree). These differences determine the specific effects of the different *CA*s on absorption, blood flux, and motility in the intestine [82].

#### Regulation of Intestinal Blood Flux, Immunity and Motility

Norepinephrine binding to α-adrenergic receptors induces vasoconstriction and increases vascular resistance, thus reducing the blood flux in the intestine. Low epinephrine levels stimulate β receptors, inducing vasodilation and consequently an increase in blood flux. However, at high levels, it induces similar effects to norepinephrine [82]. DA preferentially binds to D1 at low concentrations, and to adrenoceptors β1, and even to α1, as the DA concentrations increase. Thus, low DA levels induce vasodilation and increase blood flux, but high DA levels can induce vasoconstriction and consequently abdominal flux blood decrease.

Recently, in 2017, Mittal et al. [82], summarized the major effects of CAs in GIT:

- *Nutrient absorption*. Both epinephrine and norepinephrine play important roles in nutrient absorption regulation. Epinephrine is able to induce a hyperglycemic response acting through β-adrenergic receptors, and it increases absorption of oligopeptides when bound to α-adrenoceptors.
- *Intestinal motility.* CAs binding to β-adrenoreceptors induces smooth muscle relaxation that lead to a global food transit delay. On the contrary, their bindings to α-adrenoreceptors stimulate intestinal smooth muscle contraction, and consequently gut motility and food transit.
- *CAs, immune system and GIT.* Recently, CAs, as well as 5-HT, have been described as regulators of the innate immune system, which can be related to food intolerance. In addition, it is also reported that these amines can influence the intestinal microbiota [115].

These effects are very interesting in the context of nutrition, therefore deserving further research [116]. Fortunately, the advances of high-throughput technologies can help us to reveal the bases of these complex but important subjects for healthy and personalized nutrition.

## 5. Biochemistry and Physiology of Polyamines

### 5.1. Synthesis of Polyamines

PAs synthetized by mammalian cells are aliphatic low molecular weight polycations with 2–4 protonated amino groups at physiological pH: Put, Spd and Spm. They are essential for all living organisms from Archaea to humans, as they modulate the most basic mechanism for life, macromolecular synthesis and structure and membrane dynamics. The diamine Put is the precursor of the triamine Spd and the tetramine Spm. In mammalian cells, Put is synthetized directly from α-decarboxylation of the amino acid l-ornithine. The aminopropyl groups of Spd (1) and Spm (2) are added from decarboxylated SAM (dcAdoMet) (Figure 2d) [117]. Plants and microorganisms can synthetize other BAs with different lengths, positive charges and configurations; for instance, cadaverine (pentane-1,5-diamine), Agm, and other PAs synthetized by thermophilic microorganisms [118,119].

The first step for PA biosynthesis in mammalian cells is the hydrolysis of the guanidinium group of l-arginine catalysed by arginase (EC 3.5.3.1) activity (not shown in Figure 2d). Its product, l-ornihine, is the substrate of the PLP-dependent decarboxylase, ODC, a minor and instable protein, which is a limiting step of PA synthesis (Figure 2d). ODC product (1,4-butanodiamine) is commonly known as Put (Figure 2d). Eukaryotic ODC structure follows a model that belongs to the group IV of the PLP-dependent l-amino acid decarboxylases [7]. It needs to be a dimer to be active (≈a 102 kDa homodimer in mammals). The enzyme has one of the shortest half-lives known so far for mammalian proteins (10–50 min) and is located mainly in cytosol but it has also been detected in nucleus [120,121]. Its activity is highly regulated in response to different growth factors, oncogenes, trophic hormones, among other proliferative stimuli [122].

As mentioned before, dcAdoMet is required to synthetize higher PAs (Spd and Spm). Its synthesis involves the condensation of l-methionine and ATP to produce SAM, catalysed by any of the isoforms of *S*-adenosylmethionine synthetase or methionine adenosyltransferase (MAT, EC 2.5.1.6). SAM can then be decarboxylated by the action of *S*-adenosyl-l-methionine decarboxylase (SAMDC, EC 4.1.1.50), producing the nucleoside dcAdoMet, which acts as the aminopropyl donor for Spd and Spm synthesis (Figure 2d). SAMDC activity can also be a limiting step of the PA biosynthesis pathway. The mature enzyme suffers a post-translational maturation process, which renders the essential pyruvoyl prosthetic group [123]. Spd is synthetized by the transfer of the dcAdoMet aminopropyl moiety to the N^4^ of Put through the action of spermidine synthase (SpdS, EC 2.5.1.16). Finally, the addition of a new dcAdoMet aminopropyl moiety to the N^8^ of Spd gives rise to the tetramine Spm through the action of spermine synthase (SpmS, EC 2.5.1.22) [124].

The aminopropyl transferases SpdS and SpmS are homologus homodimers but with high substrate specificity. Steric restrictions avoid binding of Put to SpdS, as well as binding of Spd to SpmS. Nevertheless, both human enzymes contain two key Asp residues (Asp^104^ and Asp^173^ in SpdS, and Asp^201^ and Asp^276^ in SpmS), which are essential for the catalytic mechanism [119].

### 5.2. Degradation and Recycling of Polyamines

PA metabolism can be considered to be a very robust bicycle involving both metabolic branches (synthesis and degradation) (Figure 2d) [117]. Degradation involves a series of different amine oxidases. Spermine oxidase (SMO, EC 1.5.3.16) is a FAD-dependent oxidase able to directly transform Spm to Spd, 3-aminopropanal and H_2_O_2_ in the presence of H_2_O and O_2_. Several alternative splicing variants have been observed. It is highly inducible by PAs and their analogues, among other stimuli [124].

Al alternative pathway to convert Spm into Spd (as well as Spd into Put) requires the action of spermidine/spermine *N*^1^-acetyltranferase (SSAT, EC 2.3.1.57). SSAT reaction using Spm and acetyl-CoA as the substrates transforms Spm into *N*^1^-acetylspermine. This product may, in turn, follow two alternative pathways. It can be a substrate of the peroxisomal polyamine oxidase (PAO, EC 1.5.3.13) that produces the lower PA (Spd), 3-acetamidopropanal and H_2_O_2_ (Figure 2d). *N*^1^-acetylspermine can even be acetylated again in its other terminal producing *N*^1^,*N*^12^-diacetylspermine. This metabolite can be a substrate for peroxisomal PAO to produce 3-acetamidopropanal, H_2_O_2_ and *N*^1^-acetylspermidine. In a second reaction on *N*^1^-acetylspermidine, PAO produces 3-acetamidopropanal, H_2_O_2_ and Put [125,126].

SSAT also can act on Spd to form *N*^1^-acetylspermidine (and CoA). *N*^1^-acetylspermidine, which is subsequently oxidized by PAO producing 3- the diamine Put, 3-acetamidopropanal and H_2_O_2_ [125]. The diamine Put can be further degraded by DAO [118], or alternatively recycled for higher PA synthesis. Acetylated PA are more easily excreted than their deacetylated counterparts, and their levels in urine have been used as biomarkers of elevated PA metabolism [127].

In summary, the net result of the action of SMO or the tandem SSAT plus PAO is the conversion of higher PAs or their acetylated versions into their respective lower poly- or diamine, which can be again recycled in the biosynthetic pathway (Figure 2d). It conforms two energy-consuming cycles being apparently futile. However, following the metabolic control theory, “futile cycles” confer high sensitivity for modulation of metabolic pathways that need to respond to regulatory stimuli in a coordinated way as a response (sometimes a compensation) to external alterations [128]. That indeed is the case for mammalian PA metabolism, as predicted by the mathematical model of mammalian PA metabolism and proven by its further validation [117,129]. This fact explains the difficulties experienced by many experimental groups when trying to deplete intracellular PA levels as an anticancer strategy [130,131].

### 5.3. Polyamine Transport Systems

PA import systems in mammalian cells are not yet fully characterized, in spite of the multiple efforts made by different group members of the PA research community; this fact is still one of the most important handicaps to control intracellular PA levels under pathological circumstances (for instance, cancer) [132,133].

Currently, three models have been proposed and reviewed by Poulin et al. [133]. A first model proposes the action of two permeases with different locations, one located in the cytosolic inner membrane (PMPP) and an H^+^–coupled PA transporter located in vesicular membranes (VPA). A second mechanism (one step model) involves glypican-1, acting as a high affinity PA receptor. This binding could induce endocytosis leading to PA internalization, as described by Belting et al., for Spm transport [134]. The presence of NO and Ca^++^ in the endosomes would revert glypican-Spm binding. A third model proposes PA interaction with caveoline-1, which would also be reverted by NO. These mechanisms could explain the presence of higher PA in vesicles of several mammalian cell types. However, many doubts still remain concerning PA transport mechanisms through the different cellular compartments, as well as cell- and PA-specificities and regulation of each transport mechanism. Abdulhussein and Wallace recently reviewed this topic [135]. Specifically, Uemura et al., identified the amino acid transporter SLC3A2 as a Put export protein in colon cancer-derived cells [136] and also studied the specific characteristics of PA absorption by the intestinal tract, providing methods for PA transport analysis in the colon and the small intestine using membrane vesicles, culture cells, and mouse models [137].

### 5.4. Physiological Functions of Polyamines

PAs can be considered protonated amino groups kept together by aliphatic skeletons that impose specific distances among them. Thus, their positive charges and aliphatic chains can interact specifically with negative charges and hydrophobic residues/surfaces of other biomolecules (nucleic acid sequences, proteins and lipids) located at the correct distances, therefore modifying their conformations and consequently the functional properties of macromolecular structures. PAs are present and absolutely essential to keep cell viability in almost all living organisms. PA-DNA, PA-RNA and PA-membrane interactions and their conformational consequences can be reproduced and studied in vitro (in an abiotic environment) working with their purified components (PA, polynucleotides and/or lipids). This knowledge on specific molecular PA interactions gives rise to new hypotheses [138,139,140]. On the one hand, it is clear that some specific binding modes have been detected (among an immense quantity of possibilities for PA-biomolecules interactions) with physiological consequences or applications (for instance, nanotechnology applied to drug delivery) [141]. On the other hand, it is tempting to hypothesize that their interaction with both nucleic acids and membranes was of absolutely essential value from the beginning of life on Earth [142].

The interactive properties of PAs allow them to modulate a long list of processes involved in cell cycle progression and gene expression as DNA condensation, replication fidelity, RNA secondary and tertiary structure stabilization, translation initiation, elongation and fidelity, and posttranslational modification of proteins, among others [143]. The importance of the mentioned physiological functions modulated by PAs explains that their metabolism is strictly regulated, as well as very robust. Nevertheless, imbalance in PA levels is associated with a long list of human diseases that includes aberrant cell growth and differentiation and/or abnormal protein expression and folding; for instance, cancer, GIT and neurodegenerative diseases [144], as will be mentioned further on. Nevertheless, many molecular questions still remain unsolved on the molecular bases of the cellular functions of PAs [145], thus delaying the biotechnological applications of this yet unveiled knowledge.

As the other above mentioned BAs, PAs also play important roles in GIT physiology [146,147]. Synthetized de novo or uptaken from the intestinal lumen intestinal, they promote two different processes described for intestinal mucous reparation: DNA-independent cell migration and replacement of damaged cells by cell proliferation [148].

It is also proven that PAs are important for the correct biochemical and morphological maturation of intestine during the postnatal period. The benefits are dose and PA-specific (being Spm > Spd > Put). In addition, PAs have also been proposed as being involved in the correct immunological system development in neonatal intestine [146,149].

### 5.5. The Particular Case of Agmatine

Agm is a member of the PA family, as it is the product of l-arginine decarboxylation. It can be converted into Put through the action of a liver agmatinase activity, a hydrolase that removes the agmatine guanidinium moiety. It has finally been clarified that human cells express active agmatinase, but not an active arginine decarboxylase (ADC, EC 4.1.1.19) [21]. However, some bacteria present in human microbiota have, in fact, active ADC, so producing Agm that can be absorbed by intestinal epithelium. Agm protects mitochondrial functions and confers resistance to cellular apoptosis [150], being able to modulate several processes such as hepatic regeneration and renal function [151], among other proposed physiological functions. For instance, it is able to block *N*-methylaspartate receptor receptor in brain areas related to learning and memory [152], as well as modulate mental stress [153,154]. Thus, it seems to be a good candidate to participate in gut-brain axis. As HIS and Put, Agm is also a DAO substrate to produce γ-butiramide that is finally converted into γ-guanidinobutirate in CNS [39]. It can also compete with the other diamines for binding to OCT transporters [155].

## 6. Biogenic Amines and Microbiota-Intestine Crosstalk

In addition to BAs from diet and the endogenous BA metabolism of GIT cells, there is another important contributor to BA levels in the intestine: the intestinal microbiota metabolism.

On the one hand, intestinal microbiota species, like any other living organism, are able to synthesize PA [156]. These PAs can be uptaken by intestinal epithelia, thus contributing to cellular growth and tissue renewal, especially in the colon [137,156].

On the other hand, microbiota species can also synthetize any of the other BAs mentioned in this text. For instance, a PLP-dependent HDC homologous to the mammalian enzyme can be expressed by Gram negative Enterobacteria. On the contrary, Gram positive bacteria (for instance, *Lactobacillus sp.*) can express a non-homologous pyruvoil-dependent HDC [157]. The physiological effects of HIS produced by microbiota are controversial and need more research to be fully understood [15]. For instance, in spite of the general idea of a deleterious role of diet HIS on human health (see next section), it has been reported that HIS synthetized by the probiotic *Lactobacillus reuteri* acts as a positive immune regulator acting through H_2_R [158].

In addition, some bacteria potentially taking part in human microbiota can synthetize other BAs, different for the mentioned endogenous ones, and can also be bioactive for human physiology (for instance, tyramine) [159]. In general, as all BAs are described as neurotransmitters, neuroendocrine factors or neuromodulators, amines synthetized by microbiota may interact with host signals establishing a microbiota-endocrinology system crosstalk that is a part of the larger microbiota-gut-brain axis, with consequences in health and diseases [160].

Moreover, other products of the microbiota metabolism different from amines can regulate endogenous BA metabolism. For instance, bile acids and short-chain fatty acids, affect 5-HT synthesis that, in turn, directly or indirectly regulates gut motility [161] and enteric neuroimmune mechanisms [102]. A protective role of probiotics against histamine-mediated colon carcinogenesis have been recently reported [162], as well as against gastric cancer by modulating PA metabolism [163].

Summarizing, the so called microbiota-gut-brain axis is a very interesting but extremely complex open subject of current biomedicine that absolutely requires the help of new approaches from system biology and high-throughput technologies.

## 7. What Is Known about Biogenic Amines Roles in Human Gastro Intestinal Pathologies?

It is clear that alterations of elements of the BA metabolism, including transport and signalling pathways, are involved in a wide diversity of GIT pathologies. However, further efforts are needed to fully characterize the specific aberrant element(s) and/or mechanism(s) responsible for each pathological consequence. In the next subsection, we will summarize the current state of the art for the better known relationships between BA-related elements and human GIT diseases.

### 7.1. Gastric Diseases

#### 7.1.1. Peptic Ulcers

Peptic Ulcers are mucosal erosions induced by gastric secretions. In addition to those located in gastric mucosa (gastric ulcers), similar damages can appear at the entrance of duodenum (duodenal ulcers), or even in a minor percentage in the oesophagus or other intestinal segments [164]. Gastric ulcers are usually located along the minor curvature, particularly in the corpus-antrum transitional mucosa, their prevalence being higher in over 40 year-old humans. Duodenal ulcers are located between the lower part of the stomach and the start of the intestine (that is, the intestinal area exposed to gastric acid) with the highest frequency being between 20–50 year-old humans [70].

Abdominal pain is the most common symptom of peptic ulcer. Swelling, loss of appetite, nausea and/or indigestion are also usual symptoms. Associated complications include bleeding, perforation and stenosis. Bleeding is the most common complication among peptic ulcer patients (15–20%). Reciprocally, 40% of humans suffering from upper GIT bleeding are peptic ulcer patients [70].

Peptic ulcers are classified depending on their anatomical location [70,165,166]. When they are located in the stomach, they evolve as atrophic gastritis. Initially, inflammation induces parietal cell apoptosis, which in turn induces gastric acid hyposecretion and mucosal atrophy. Chronic inflammation and mucosal atrophy lead to gastric ulcers and eventually to gastric cancer.

Other individuals present gastritis located in the pylorus area. In this case, excessive quantities of gastric acid are usually produced, which lead to duodenal ulcers. The inflammatory response induces cytokine secretion that finally induces dysregulation of endocrine cells located in this area. Thus, G cells are stimulated to overproduce gastrin, while somatostatin secretion is inhibited. As a consequence, HIS synthesis and secretion increase, which in turn promotes proliferation and stimulation of parietal cells, leading to gastric acid hypersecretion (Figure 3). These facts explain the treatment of the ulcers with proton pump inhibitors and H_2_R antagonists [167,168].

Infection by *Helicobacter pylori* is a very common origin of peptic ulcers. Nevertheless, other risk factors as alcohol and smoking abuse, as well as a continual use of several drugs (for instance, nonsteroidal anti-inflammatory drugs or NSAIDs) have been described. *H. pylori* is a microaerophilic flagellated bacteria able to colonize human gastric mucosa. It is a highly common infection that can take place for tens of years. This Gram-negative bacteria is considered an important pathogenic agent associated with several human pathologies like chronic gastritis, gastrointestinal ulcers, and neoplasms such as gastric adenocarcinomas and gastric mucosa-associated lymphomas (Table 4) [169].

*H. pylori* induces HDC expression, and consequently an increase in endogenous HIS synthesis, which leads to an inflammatory response including an increased presence of neutrophils and lymphocytes, which in turn produces different cytokines and chemokines (for instance, IL-1, IL-6, TNF-α and IFN-γ). Gene polymorphisms detected in some of these human cytokine genes are proposed as being involved in resistance or susceptibility to *H. pylori* [75,170].

Different signalling mechanisms have been described to explain the pathological consequences of *H. pylori* infection. On the one hand, there is a CAG (cytotoxin-associated gene)-dependent pathway (involving signal transduction elements like Rho GTPases, PKA, MKK4 and JNK, and the transcription factors AP-1 and NF-κB), which result in synthesis and secretion of cytokines acting as an innate immune response. On the other hand, MAP kinase pathway (involving Raf-1, ERK, MEK) is activated by a CAG-independent mechanism, which finally results in activation of BP1 and BP2. These transcription factors act as inducers on the HDC promoter, which lead to an increase in gastric acid synthesis [171].

#### 7.1.2. Gastric Cancer

Gastric cancer presents a high morbidity and mortality, being described as one of the most common worldwide malignant neoplasms. Nevertheless, its prevalence has decreased in the last decades in most European countries probably due to changes in lifestyle such as smoking reduction and *H. pylori* control [172].

Chronic inflammation produced by the pathogen can result in changes in the normal architecture of gastric mucosa, destruction of gastric glands, parietal cell loss, decreased gastric acid secretion, intestinal metaplasia, and finally gastric cancer. Thus, World Health Organization estimates that chronic *H. pylori* infection increases the risk of gastric cancer by 10 times, considering it as a class I carcinogen [70]. In fact, a reduced gastric acid secretion is not only a predisposition to gastric ulcers, but also to gastric cancer. Low levels of gastric acid reduce vitamin C absorption and allow an excessive growth of salivary and intestinal bacteria in the stomach, which can promote carcinogenesis [165].

It has been observed that treatments with gastrin/CCK-2 receptor antagonists reduce parietal cell apoptosis and inhibit gastric atrophy. Similar results were obtained with an irreversible H_2_R antagonist (i.e., cimetidine); suggesting that both receptors could be involved in gastric cell apoptosis and carcinogenesis [173]. However, a prospective, double blind trial carried out among hundreds of gastric cancer patients by the British stomach cancer group did not result in any increase of survival [174].

Recent research on H_4_R physiological roles reveals interesting information on the involvement of the receptor in the relationship between the immune system and GIT carcinogenesis [70].

As PAs (mainly Spd and Spm) are also essential for gastric cancer progression, some authors claim that new probiotic-based anticancer strategies are able to reduce endogenous PA levels and gastric cancer growth as a result [163].

### 7.2. Intestinal Diseases

#### 7.2.1. Irritable Bowel Syndrome

Formerly known as spastic colon, irritable bowel syndrome (IBS) is a prevalent disorder that is characterized by alterations in intestinal secretions and motility, mainly affecting the colon. The symptoms (cramps, abdominal pain, intestinal habit alterations, and food intolerances) can appear during childhood or in adults [175,176]. Different subtypes have been described. The IBS-D subtype is characterized by diarrhoea; IBS-C is characterized by constipation; and IBS-M presents both intestinal alterations [175].

Several research groups have proposed the involvement of 5-HT-related elements (enzymes, transporters and receptors) in the pathology. For instance, results of several studies point out genetic variants of the gene encoding the 5-HT transport SERT (chromosome 17) that could predispose to IBS [177]. However, other results are not conclusive and sometimes contradictory [178]. Thus, it is one of the complex BA-GIT relationships needing further investigation.

Different treatments are prescribed for IBS patients depending on the severity of the symptoms. Diet adjustments and behavioaral education are usually enough for mild and moderate symptoms. However, for most severe forms, multidisciplinary approaches including pharmacotherapy are required. For IBS-D, treatment with antidiarrhoeal drugs, such as loperamide, can be necessary. In the case of women with severe symptoms, the 5-HT_3_R antagonist alosetron can be used while the 5-HT_4_R agonist prucalopride is used to relieve constipation in IBS-patients [179].

Other drug discovery initiatives are trying to develop effective inhibitors of tryptophan hydroxylase 1 (TPH1, the isoform expressed in GIT), which are unable to pass the blood-brain barrier, as an alternative to reduce 5-HT synthesis by EC cells, and consequently reduce/avoid their deleterious effects induced by dysregulation of the gastrointestinal serotonergic system (for instance IBS and carcinoid syndrome) [180,181].

#### 7.2.2. Inflammatory Bowel Diseases

Crohn disease (CD) and ulcerative colitis (UC) are inflammatory bowel diseases (IBD) with different characteristics. Nevertheless, both diseases occur with alternating periods of remission and relapse. UC is characterized by inflammation with the presence of superficial colon mucosa ulcers that usually originate in the colon and then progress towards upper colon sections. UC symptoms include diarrhoea, cramping, and rectal bleeding. CD is characterized by a discontinuous pattern along the intestinal tract and can present larger ulcerations and sometimes granulomas; abdominal pain, diarrhoea, weight loss and bleeding being its most common symptoms [45].

Different research groups have observed a HIS secretion increase in jejunum of CD patients and high HIS levels in the intestinal mucosa of UC patients. In addition, levels of the HIS degradation product *N*-methylhistamine are elevated in the urine of both disease patients, which suggest a more active HIS metabolism (synthesis and degradation) in the intestine with respect to control individuals. The results indicate that degranulation of mast cells infiltrated along the intestinal tract must be involved in these diseases. However, there is a lack of information about molecular details of the signalling mechanisms responsible for the HIS effects on IBD evolution, thus blocking the development of efficient intervention strategies [75,182]. It has been proven that the chronic use of H_2_R receptor antagonists increases the risk of more severe CD, suggesting a protective role of HIS on the intestinal mucosa when acting through an H_2_R receptor [45]. IBD intervention acting through H_4_R receptor has also been recently proposed [183,184]. In fact, several studies highlight the potential of H_4_ receptor targeted therapy in the treatment of various gastrointestinal disorders such as IBD, IBS and cancer [184].

DAO has been suggested as an IBD marker, but it is a controversial subject [64,185]. Currently, IBD treatment consists of diet adjustment, psychological support, and eventually surgery. Recently, it has been proven that microbiota is usually altered in IBD patients. Working with a mouse model of intestinal inflammation, it has been demonstrated that the probiotic *Lactobacillus reuteri* is able to reduce intestinal inflammation by a HIS signalling-dependent mechanism, thus acting as a preventive factor for cancer risk associated with chronic inflammation [186]. Microbiota can also have a positive role in IBD patients, as microbial-derived metabolites (for instance, bile acids and short-chain fatty acids) can regulate intestinal 5-HT synthesis, and consequently intestinal motility, which opens new perspectives for probiotic-based strategies [161].

#### 7.2.3. Intestinal Neoplasias

Colon cancer is considered the most common GIT cancer. It is a multifactorial disease and its etiology can combine genetic inherited factors, exposition to environmental risk factors (including diet), as well as other endogenous circumstances such as chronic intestinal inflammation. At present, it is one of the most frequent human cancers, besides being one of the principal causes of death among human cancer patients [187]. As in other cancer types, colon cancer biopsies present increased activities of PA-synthesis key enzymes and elevated PA content up to 10-15-fold with respect to the levels observed in normal colon epithelium. Thus, together with inflammation, PAs are considered to be markers of colon carcinogenesis [188].

It is known that several oncogenes and suppressor genes that regulate specific phases of colon carcinogenesis are also involved in PA metabolism regulation. Under normal conditions, the suppressor gene adenomatous polyposis coli (APC) repress MYC transcription, a family of transcription factors required for cell proliferation. MYC overexpression is related to uncontrolled proliferation and progression of carcinogenic process in different cancer types, including colon cancer. Members of MYC family are inducers of ODC transcription [189]. In addition, APC up-regulate the expression of ODC antizymes, a protein family acting as ODC inhibitors, as they bind to ODC monomer blocking the active quaternary conformation of the enzyme, thus targeting the ODC monomer for an antizyme-dependent and ubiquitin-independent proteasomal degradation. When APC is deleted or inactivated by mutation during the early stages of carcinogenesis (as occurring in adenomatous polyposis patients), MYC APC-induced repression is lost, and consequently ODC and PA synthesis are upregulated. In addition, the upregulation of ODC antizyme expression is lost, which leads to an increase in ODC turnover [190].

Active KRAS oncogene downregulates the peroxisome proliferator-activated receptor gamma (PPARγ), which has been proposed as a marker for colorectal cancer survival [191]. Transcription of the key enzyme for PA degradation (SSAT) is upregulated by PPARγ response elements (PPREs) present in SSAT promoter [192]. In advanced stages of colon cancer, oncogenic mutations in KRAS lead to permanent KRAS activation, which in turn downregulates PPARγ and, consequently, SSAT expression and PA degradation [193]. These regulatory mechanisms explain the molecular bases of the relationship between oncogenic events and PA elevation in colon cancer patients. As mentioned in previous sections, elevation of PA levels helps replication and macromolecular synthesis of the transformed cells and confers other advantages for cancer progression.

In addition to the endogenous regulation of PA metabolism, it is also worth mentioning that diet and microbiota are also potential PA sources [194]. The lack of effective PA absorption inhibitors, as well as the robustness of PA metabolism, is blocking the success of antitumoural strategies based on PA depletion in different cancer types [190]. In colon cancer, treatment depends on the progression stage. Surgery can be enough during the first stages; then, chemotherapy or immunotherapy must be required. What about chemoprevention? The irreversible ODC inhibitor DFMO is able to act on both Enterobacteria and mammalian ODC activities. As PAs are essential for colon cancer progression, DFMO could be effective as a chemopreventive agent for putative familiar colon cancer patients. This was the hypothesis claimed by Gerner and Meyskens several years ago, and validated with positive results when administered as a combined therapy with NSAIDs, the latest acting as PPARγ inducers [188,190].

Working with HDC knocked out mice under treatment with probiotics (*Lactobacillus reuteri*), results obtained by Gao et al. [162] indicate that luminal HIS produced by gut microbiota could suppress inflammation-associated colon carcinogenesis.

As mentioned above (Table 1), HIS is an immune system regulator playing important roles in immune cell development, span lives, and functions. These effects can involve any of the four receptors (mainly H_1_R and H_4_R) [37,195]. HIS has also been described as a cell proliferation regulator of several cancer types (for instance, breast cancer, GIT cancer, leukaemia, lung cancer, lymphoma) [196]. These effects can be different depending on the HIS receptors expressed by the different cell types. In addition, communication between the immune system and cancer is a dynamic process involving different immune cells; for instance, macrophages, monocytes, mast cells and regulatory B cells and T cells [197,198]. Consequently, HIS effects on carcinogenesis and cancer progression is a very complex but interesting topic, which also deserves further research efforts. Recent results support the therapeutic potential of H_4_R ligand in several cancer types, including colon cancer [198].

Evolution of Zollinger-Ellison syndrome, a rare GIT pathology caused by the presence of gastrin-secreting tumours in pancreas and/or intestine, involves both synthesis and secretion of HIS [29].

## 8. Conclusions and Future Prospects

Previous sections demonstrate that BAs play important physiological roles in the entire GIT. Consequently, aberrant functions of the metabolic pathways are involved in the most important gastrointestinal pathologies. HIS metabolism seems to be mainly important in gastric physiopathology, as well as in inflammatory intestinal diseases. 5-HT plays major pathophysiological roles in the intestine, as an immune modulator and regulator of the intestinal smooth muscle contraction/relaxation. Its involvement in inflammatory diseases needs further clarification. CAs are modulators of intestinal absorption, blood flux and motility and they have also been proposed as immune modulators in the GIT system. Some of these functions may be regulated by 5-HT [104]. This is a very interesting hypothesis that would also require more research efforts to be fully validated. PAs, being essential biomolecules for cell growth, are important for both epithelial reparation and proliferation. Thus, they are beneficial for a healthy intestinal epithelium and have been described as both immune and epithelial permeability modulators in GIT [199], but also proposed as a promising target for colon cancer chemoprevention. From a phenomenological point of view, all B/A-BAs have been described as modulators of both immunity and epithelial cell growth in the GIT, but many of the underlying molecular mechanisms are not fully characterized, yet. In any case, these results point to the GIT as an interesting scenario to study BA metabolic and functional interplay.

In spite of the specialization of the amine effects along the different segments of the GIT, in some cases, their metabolic routes are coincident in a given GIT segment, so they can share/compete for common elements, thus establishing a crosstalk among their metabolism and consequently their physiological missions. For instance, enzymes such as decarboxylases (i.e., DDC), amine oxidases (i.e., MAO and DAO), cofactors (i.e., PLP, BH_4_), and metabolites (i.e., SAM), among others. Moreover, at least in neurons, heteroreceptor complexes have also been detected among HIS and DA receptors [200], and both Spd and HIS are ligands and modulate *N*-methylaspartate receptor activity [201]. In mouse mast cells, synthesis of PA, HIS and 5-HT seem to be antagonistic processes in the mast cell differentiation process. Thus, the pathophysiological consequences of this cross-talk among BA metabolic elements still present many gaps and open questions (mentioned throughout this review) in the GIT context, which deserve deeper molecular characterization, as this information could provide valuable insights useful for new diagnosis and intervention initiatives in the gastroenterology field as well as more personalized nutritional advices and preventive actions.

It is clear that the pathophysiological effects of B/A-BAs in GIT is a very complex issue that interacts with and is modified by a very extensive list of endogenous and exogenous factors, from metabolic interactions with other immune and/or neuroendocrine compounds/systems to the influx of diet and microbiota composition. The full characterization of the entire involved interactions still requires filling many gaps on specific biochemical and molecular details. This research objective should be helped by systemic approaches to provide integrative views (and even predictive models) of the multiple pathophysiological processes associated with BAs in the GIT. In fact, several independent groups have proposed to approach the problem, or subsets of the problem, by using integrative high-throughput and Systems Biology strategies currently successfully used with many other complex biological systems [1,202].

The translational benefits of the final objective are clear taking into account that the topic involves three of the most important but complex physiological systems for a human being, neurotransmission/neuroendocrine, immune and digestive systems. Consequently, life quality and/or span life of many human beings can depend on research advances in the topic. Thus, it should be considered among the research priorities not only for nutrition but for biomedicine, in general. In addition, different companies have developed a wide spectrum of drugs capable of modulating different elements of BA metabolism and signalling. The usefulness and/or efficiency of these compounds (or their analogues/derivatives) will probably increase when a deeper degree of integrative knowledge about the molecular basis and the roles of all B/A-BAs in the GIT system is achieved.

## Figures and Tables

**Table 1:** Nomenclature, precursors and main functions of the basic and aromatic amines involved in the gastrointestinal pathophysiology.

Common Names (Abbreviations) *	IUPAC Names	Precursor l-Amino Acids	Physiological Roles
Histamine (HIS)	2-(1H-Imidazol-4-yl)ethanamine	l-Histidine	Neurotransmitter. Immune mediator. Gastric acid secretion inducer.
Serotonin (5-HT)	3-(2-Aminoethyl)-1H-indol-5-ol	l-Triptophan	Neurotransmitter related to reward motivated behaviour. Modulator of vessel constriction and intestinal motility.
**Catecholamines (CAs):**		l-Tyrosine	Blood pressure regulators. Modulators of nutrient absorption and intestinal motility.
Dopamine (DA)	4-(2-Aminoethyl)benzene-1,2-diol		
Epinephrine	(R)-4-(1-Hydroxy-2-(methyl amino)ethyl)benzene-1,2-diol		
Norepinephrine	(R)-4-(2-amino-1-hydroxy ethyl)benzene-1,2-diol		
**Polyamines (PAs):**			Essential for cell viability, proliferation and correct differentiation.
Putrescine (Put)	Butane-1,4-diamine	l-Ornithine	
Spermidine (Spd)	*N*′-(3-aminopropyl)butane-1,4-diamine	l-Ornithine + l-Methionine	
Spermine (Spm)	*N*,*N*′-bis(3-aminopropyl)butane-1,4-diamine		
Agmatine (Agm)	2-(4-aminobutyl)guanidine	l-Arginine	Anti-apoptotic effects. Positive effects on brain, hepatic and renal functions.

Data from references [16,17,18,19,20,21,22,23,24]. *, Abbreviations used in the text.

**Table 2:** Molecular and functional properties of human histamine receptor types.

Properties	HIS Receptor 1 (H_1_R)	HIS Receptor 2 (H_2_R)	HIS Receptor 3 (H_3_R)	HIS Receptor 4 (H_4_R)
Chromosome	3	5	20	18
Molecular weight (KDa)	56	40	49	44
G protein signalling	Gα_q_	Gα_s_	G_i/o_	G_i/o_
Elicited signalling	PLC activation Increase of Ca^2+^ Production of NOS and cGMP	PKA activation Increase of cAMP PLC activation Increase of Ca^2+^	Decrease of cAMP Inhibition of Ca^2+^ channels	Inhibition of cAMP Stimulation of MAP kinase phosphorylation
Expression	Brain, smooth muscle, skin, gastrointestinal and genitourinary tract, adrenal medulla, immune system and heart	Brain, smooth muscle, skin, gastrointestinal and genitourinary tract, adrenal medulla, immune system and heart	Widely found in brain and gastric mucosa	Inflammatory cells, dendritic cells and peripheral nerves
Physiological effects	Smooth muscle contraction Vasodilation and increase of vascular permeability	Inhibition of chemotaxis in basophils, gastric secretion of HCl and duodenal bicarbonate secretion	Release regulation of HIS (and other neurotransmitters) release from neurons Inhibition the secretion of gastric acid	Inflammatory processes such as allergies and asthma

Data from references [16,17,18]. Abbreviations; cAMP, 3′-5′-cyclic adenosine monophosphate; cGMP, 3′-5′-cyclic guanosyl monophosphate; HIS, histamine; MAP, mitogen-activated protein; NOS, nitric oxide synthase; PLC, phospholipase C.

**Table 3:** Molecular and functional properties of the best-known human 5-HT receptor subtypes important for GIT functions.

Properties	5-HT_1A_ Receptors (5-HT_1A_R)	5-HT_1D_ Receptors (5-HT_1D_R)	5-HT_2_ Receptors (5-HT_2_R)	5-HT_3_ Receptors (5-HT_3_R)	5-HT_4_ Receptors (5-HT_4_R)	5-HT_7_ Receptors (5-HT_7_R)
Chromosome	5	6	13/2/X	11 (A, B and C) and 3 (D and E)	5	10
Molecular weight (KDa)	421	390	471/481/458	Pentameric 478 (A); 441 (B); 447 (C); 279 (D); 471 (E)	387	445
G protein signalling	G_i/o_	G_i/o_	G_q/11_	Activated by ligand binding and opening channels	G_s_	G_s_
Expression	Enteric neurons, substantia nigra, hippocampus	Enteric neurons, substantia nigra, basal ganglia	Stomach, fundus, caudate nucleus, cerebellum	Enteric, sympathetic and vagus nerves, area postrema	Enteric neurons (myenteric plexus), hippocampus	Smooth muscle, thalamus, hypothalamus and hippocampus
Physiological effects	Neuronal inhibition	Neuronal inhibition	Muscle contraction	Neuronal depolarization Increased neurotransmitter release	Muscle contraction Positive effects on cholinergic transmission.	Muscle relaxation

Data from references [95,103].

**Table 4:** Features associated with *H. pylori* infections and subsequent inflammation located in different parts of the gastrointestinal tract.

Location	Acid Secretion	Gastric Features and Histology	Intestinal Features and Histology	Pathology
Stomach (pan-gastritis)	Hyposecretion	Chronic inflammation and parietal cell apoptosis Atrophy Intestinal metaplasia	Normal	Gastric ulcer Gastric cancer
Pylorus area	Hypersecretion	Chronic inflammation and increased gastrin released Inhibition of somatostatin Increase parietal cell stimulation	Gastric metaplasia Active chronic inflammation	Duodenal ulcer

Data from reference [16,17,18].
